# Contribution of Caspase(s) to the Cell Cycle Regulation at Mitotic Phase

**DOI:** 10.1371/journal.pone.0018449

**Published:** 2011-03-30

**Authors:** Toshiaki Hashimoto, Ushio Kikkawa, Shinji Kamada

**Affiliations:** Biosignal Research Center, Kobe University, Nada-ku, Kobe, Japan; University of Chicago, United States of America

## Abstract

Caspases have been suggested to contribute to not only apoptosis regulation but also non-apoptotic cellular phenomena. Recently, we have reported the involvement of caspase-7 to the cell cycle progression at mitotic phase by knockdown of caspase-7 using small interfering RNAs and short hairpin RNA. Here we showed that chemically synthesized broad-spectrum caspase inhibitors, which have been used to suppress apoptosis, prevented the cell proliferation in a dose-dependent manner, and that the subtype-specific peptide-based caspase inhibitor for caspase-3 and -7, but not for caspase-9, inhibited cell proliferation. It was also indicated that the BIR2 domain of X-linked inhibitor of apoptosis protein, functioning as an inhibitor for caspase-3 and -7, but not the BIR3 domain which plays as a caspase-9 inhibitor, induced cell cycle arrest. Furthermore, flow cytometry revealed that the cells treated with caspase inhibitors arrested at G_2_/M phase. By using HeLa.S-Fucci (*f*luorescent *u*biquitination-based *c*ell *c*ycle *i*ndicator) cells, the prevention of the cell proliferation by caspase inhibitors induced cell cycle arrest at mitotic phase accompanying the accumulation of the substrates for APC/C, suggesting the impairment of the APC/C activity at the transition from M to G_1_ phases. These results indicate that caspase(s) contribute to the cell cycle regulation at mitotic phase.

## Introduction

Caspase is a family of cysteine proteases that are required for cytokine maturation and apoptosis execution [1]–[3]. In mammals, caspase-1, -4, and -5, which have a large prodomain and cleave the procytokines, are referred to as inflammatory caspases. Another group of caspases with a large prodomain including caspase-2, -8, -9, and -10 are termed as the initiator caspases of apoptosis, whereas the enzymes with a short prodomain comprising caspase-3, -6, and -7 are the effector caspases of apoptosis. The effector caspases are activated through proteolytic processing catalyzed by the initiator caspases. On the other hand, the inhibitor of apoptosis proteins (IAPs) are identified as the endogenous inhibitors for apoptosis, and eight IAPs have been thus far isolated in mammals [4], [5]. They share a conserved region known as the baculovirus IAP repeat (BIR) domain. XIAP is one of IAPs, which contains three distinct BIR domains and a RING finger domain, and binds directly to caspase-3, -7, and -9 to inhibit their protease activity.

Cell cycle is controlled by the ubiquitin-mediated proteolysis of the key regulators [6]–[9]. Two major classes of ubiquitin ligases, the SKP1-CUL1-F-box-protein (SCF) complex and the anaphase-promoting complex/cyclosome (APC/C), play central roles in the cell cycle regulation through the ubiquitination of various cell cycle regulators. The SCF complex functions from late G_1_ phase to early M phase, whereas APC/C is active from anaphase to the end of G_1_ phase. There are two activators for APC/C, cell division cycle 20 (Cdc20) and Cdh1, which bind with APC/C and recognize respective substrate proteins. APC/C^Cdc20^ targets securin and cyclin B1 for their destruction, and promotes sister chromatid separation. APC/C^Cdh1^ facilitates exit from M phase and maintains G1 phase by mediating the degradation of a variety of substrates including PLK1, cyclin B1, and geminin.

Recently, it has been proposed that initiator and effector caspases of apoptosis are required for non-apoptotic functions [10]–[15]. Concerning caspase-3, an active caspase-3 fragment is immunostained in the proliferative region in rat brain [16], caspase-3 is upregulated just prier to mitosis [17], and the treatment with a caspase-3 inhibitor induces cell death at late mitosis [18]. Caspase-8 is pointed out to play an essential role in the development and activation of immune cells [15], [16]. In addition, the activity of caspase-3, -7, -8, and -9 is elevated in some tumor cells [19]–[21]. We have recently reported a possible involvement of caspase-7 to the cell cycle progression at mitosis [22]. The clear evidence, however, has not been available for the role of caspases in the regulation of cell proliferation.

In the present study, we showed the contribution of caspase(s) to the regulation of cell proliferation by the application of the peptide-based caspase inhibitors and the expression of the BIR domains of XIAP in the cultured mammalian cancer cells. Furthermore, it was shown that the inhibition of cell proliferation by caspase inhibitors was induced by the failure of the APC/C regulation at the transition from M to G_1_ phases.

## Results

### Peptide-based broad-spectrum caspase inhibitors prevented cell proliferation

**Figure 1:**
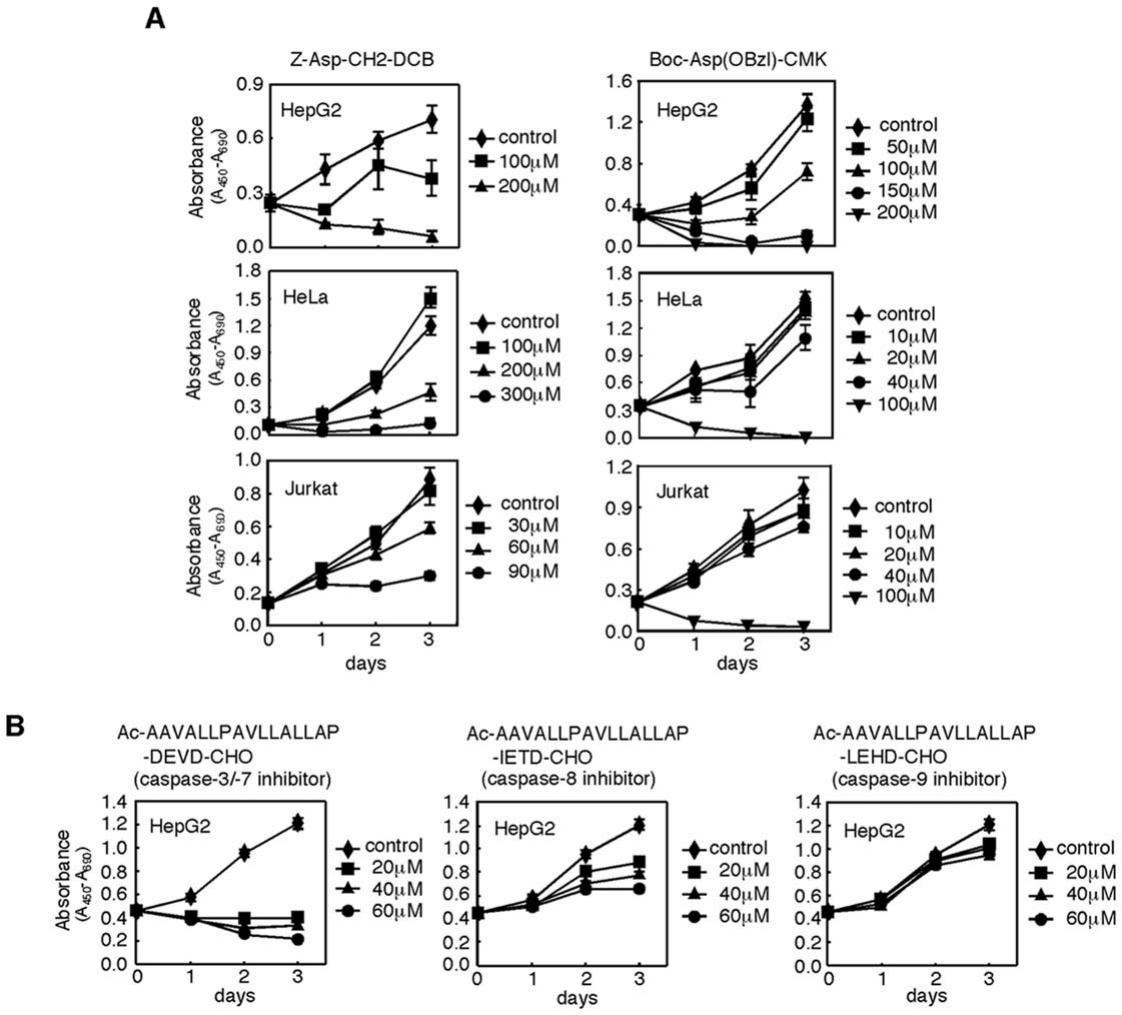
Peptide-based caspase inhibitors prevent cell proliferation. **A**) The effects of broad-spectrum caspase inhibitors on cell proliferation. HepG2, HeLa, and Jurkat cells were cultured in the presence of broad-spectrum caspase inhibitors as indicated. **B**) The effects of subtype-specific caspase inhibitors on cell proliferation. HepG2 cells were cultured in the presence of subtype-specific caspase inhibitors as indicated. Cell viability was determined by WST-1 assay. DMSO-treated cells were used as a control.

Caspases are pointed out to contribute to the regulation of cell proliferation [10], [11], [15], and we have recently reported that caspase-7 has a novel function in the progression of mitosis by using small interfering RNAs (siRNAs) and short hairpin RNA (shRNA) directed towards caspase-7 [22]. It has been unclear, however, whether the inhibition of caspase activities prevents cell proliferation. Therefore, we examined the effects of peptide-based caspase inhibitors on cell proliferation. Cell-permeant broad-spectrum caspase inhibitors, Z-Asp-CH_2_-DCB and Boc-Asp(Obzl)-CMK, are dissolved in DMSO to be added to the culture medium. We first determined the highest concentration of DMSO that could be added to the culture medium without affecting proliferation, since DMSO is toxic to cells. DMSO at 3% had no significant effect on cell proliferation of HepG2 cells and the compound at 0.6% was inert for HeLa and Jurkat cells (data not shown). Therefore, DMSO at these concentrations was used for each cell line as a vehicle for the reagents in the following experiments. Broad-spectrum caspase inhibitors prevented proliferation of HepG2, HeLa, and Jurkat cells in a dose-dependent manner, with complete inhibition at the highest concentrations (Figure 1A), suggesting that caspase activities are required for proliferation in HepG2, HeLa, and Jurkat cells.

Next, we set out to clarify which caspase is essential for cell proliferation by using subtype-specific cell-permeant caspase inhibitors, in which each inhibitor peptide was fused to the hydrophobic region of the signal peptide of Kaposi fibroblast growth factor (K-FGF) at its N-terminal end to confer cell permeability: Ac-AAVALLPAVLLALLAP-DEVD-CHO (caspase-3 and -7 inhibitor), -IETD-CHO (caspase-8 inhibitor), and -LEHD-CHO (caspase-9 inhibitor). The inhibitor for caspase-3 and -7 was most effective for preventing proliferation of HepG2 cells (Figure 1B). The caspase-8 inhibitor also attenuated proliferation of HepG2 cells but less effectively than the caspase-3 and -7 inhibitor, and the caspase-9 inhibitor had only a marginal effect. It is less possible that the inhibition of cell proliferation is due to the toxic effect of the signal peptide of K-FGF, because the caspase-9 inhibitor having the peptide did not show significant growth suppression. The results employing the peptide-based caspase inhibitors indicate that caspase-3 and/or caspase-7 plays an essential role in cell proliferation, and that caspase-8, but not caspase-9, may function as an upstream caspase of caspase-3 and/or -7.

### Overexpression of XIAP-BIR2, but not XIAP-BIR3, prevented cell proliferation

**Figure 2:**
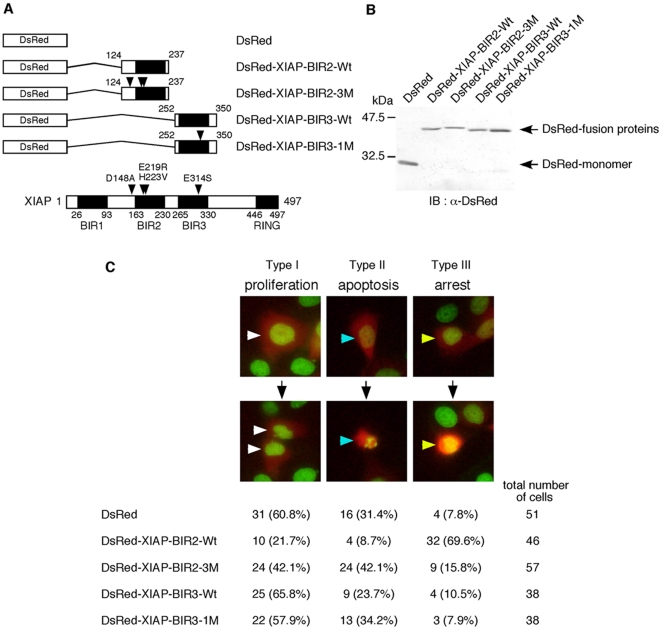
Overexpression of XIAP-BIR2, but not XIAP-BIR3, inhibits cell proliferation. **A**) Diagrams showing the constructions of DsRed-XIAP-BIRs expression plasmids and the structure of XIAP with the positions of the point mutations. Mutated amino acid residues are indicated by arrowheads. **B**) Expression of DsRed and DsRed-XIAP-BIRs fusion proteins in 293T cells. 293T cells were transiently transfected with DsRed or each DsRed-XIAP-BIRs expression plasmid as indicated. At 18 hour after transfection, cells were harvested and lysates were subjected to immunoblot analysis with the anti-DsRed antibody. **C**) The effects of overexpression of DsRed-XIAP-BIRs on cell proliferation. HeLa-H2B-GFP cells were seeded at a density of 1×10^5^ cells per 35-mm glass bottom dish and incubated for 12 hours. Cells were transfected with DsRed or each DsRed-XIAP-BIRs expression plasmid as indicated and further incubated for 24 hours. Then, cells were observed with the time-lapse fluorescence microscope at 15-minute intervals for 24 hours (see Videos S1, S2, S3, S4, S5). The observation indicated that DsRed-positive cells were divided into three types of cells; type I, normally divided into two nuclei showing proliferation (white arrowheads); type II, condensed and fragmented nuclei showing apoptotic cells (blue arrowheads); type III, condensed but not fragmented nuclei showing proliferation-arrested cells (yellow arrowheads). The cell numbers of each type and the percentages to the total DsRed-positive cells are shown.

To further explore the role of caspases in cell proliferation, we examined the effects of XIAP, which inhibits caspase activities through a different mode of action from that of the peptide-based inhibitors [23], [24]. XIAP has been suggested to inhibit apoptosis through mechanisms independent of its binding ability to caspases [25]–[27], but, in practice, the BIR2 domain with the linker region (amino acids 124 to 237) and the BIR3 domain (amino acids 252 to 350) suppress the protease activity of caspase-3 and -7 [28] and caspase-9 [24], respectively. Therefore, we first established a monitor system to visualize the recombinant XIAP protein as well as chromatin by transiently introducing XIAP fused to DsRed-monomer in HeLa-H2B-GFP cells stably expressing histone H2B-GFP fusion protein [29]. The expression of XIAP and the structure of chromatin were monitored as the fluorescence of DsRed and GFP, respectively, by time-lapse fluorescence microcopy (Figure 2 and Video S1). To make use of XIAP as the subtype-specific caspase inhibitors in relation to cell proliferation, we constructed the BIR2 domain with the linker region fused to DsRed-monomer (DsRed-XIAP-BIR2-Wt) and the BIR3 domain fused to DsRed-monomer (DsRed-XIAP-BIR3-Wt) as a caspase-3 and -7 inhibitor and caspase-9 inhibitor, respectively (Figures 2A and B). DsRed-expressing cells were classified into three types during the observation for 24 hours (Figure 2C and Videos S1, S2, S3, S4, S5): type I cells, which divided normally into two nuclei, representing proliferating cells; type II cells, which had condensed and fragmented nuclei, representing apoptotic cells; type III cells, which had condensed but not fragmented nuclei, representing proliferation-arrested cells. Cells overexpressing DsRed-monomer had 60.8% type I, 31.4% type II and 7.8% type III cells, indicating that more than half of the cells expressing DsRed were proliferating and that DsRed itself has only a weak cytotoxicity. The expression of DsRed-XIAP-BIR2-Wt and DsRed-XIAP-BIR3-Wt decreased apoptosis, 8.7 and 23.7% type II cells, possibly through inhibition of caspase-3 and -7 and caspase-9 activities, respectively. Importantly, the cells expressing DsRed-XIAP-BIR2-Wt showed a decrease of the proliferating cells (type I) and an increase of proliferation-arrested cells (type III), 39.1 and 61.8% differences with that of the cells expressing DsRed, respectively. The expression of DsRed-XIAP-BIR3-Wt, however, had little effect on cell proliferation. It is reported that the mutants of XIAP, D148A/E219R/H223V in BIR2 [28] and E314S in BIR3 [30], cannot inhibit caspase-3 or -7 and caspase-9, respectively. These mutations abolished not only the inhibition of apoptosis but also the changes of the numbers of the proliferating and proliferation-arrested cells in transfected HeLa-H2B-GFP cells. These results indicate that the activity of caspase-3 and/or caspase-7, but not caspase-9, is necessary for cell proliferation, which are consistent with those obtained by the cell-permeant peptide-based caspase inhibitors as shown in Figure 1B.

### Peptide-based caspase inhibitor induced cell cycle arrest at G_2_/M phase

**Figure 3:**
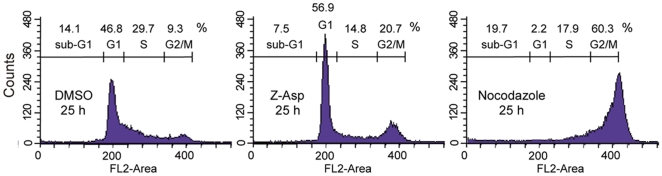
Peptide-based caspase inhibitor induces cell cycle arrest. HeLa cells were cultured in the presence of 0.6% DMSO, 300 µM Z-Asp-CH_2_-DCB, or 0.1 µg/ml nocodazole for 25 hours, and cell cycle profiles were observed by flow cytometric analysis.

To clarify the roles of caspase(s) in the cell cycle progression, the flow cytometric analysis was carried out using HeLa cells treated with the caspase inhibitor. HeLa cells treated with DMSO, Z-Asp-CH_2_-DCB, or nocodazole for 25 hours were analyzed with flow cytometry to determine the cell cycle profiles (Figure 3). Although most of nocodazole-treated cells were accumulated in M phase, consistent with the ability of nocodazole to disrupt microtubules, the caspase inhibitor-treated cells showed an increase of cells at G_1_ and G_2_/M phases as compared with the DMSO-treated cells. These results suggest that caspase inhibition induces cell cycle arrest at G_1_ and G_2_/M phases, and that caspase(s), that is necessary for the cell cycle progression, plays a role, at least in part, around G_2_/M phase.

### Peptide-based caspase inhibitor impaired the transition from M to G_1_ phases through inhibiting APC/C

**Figure 4:**
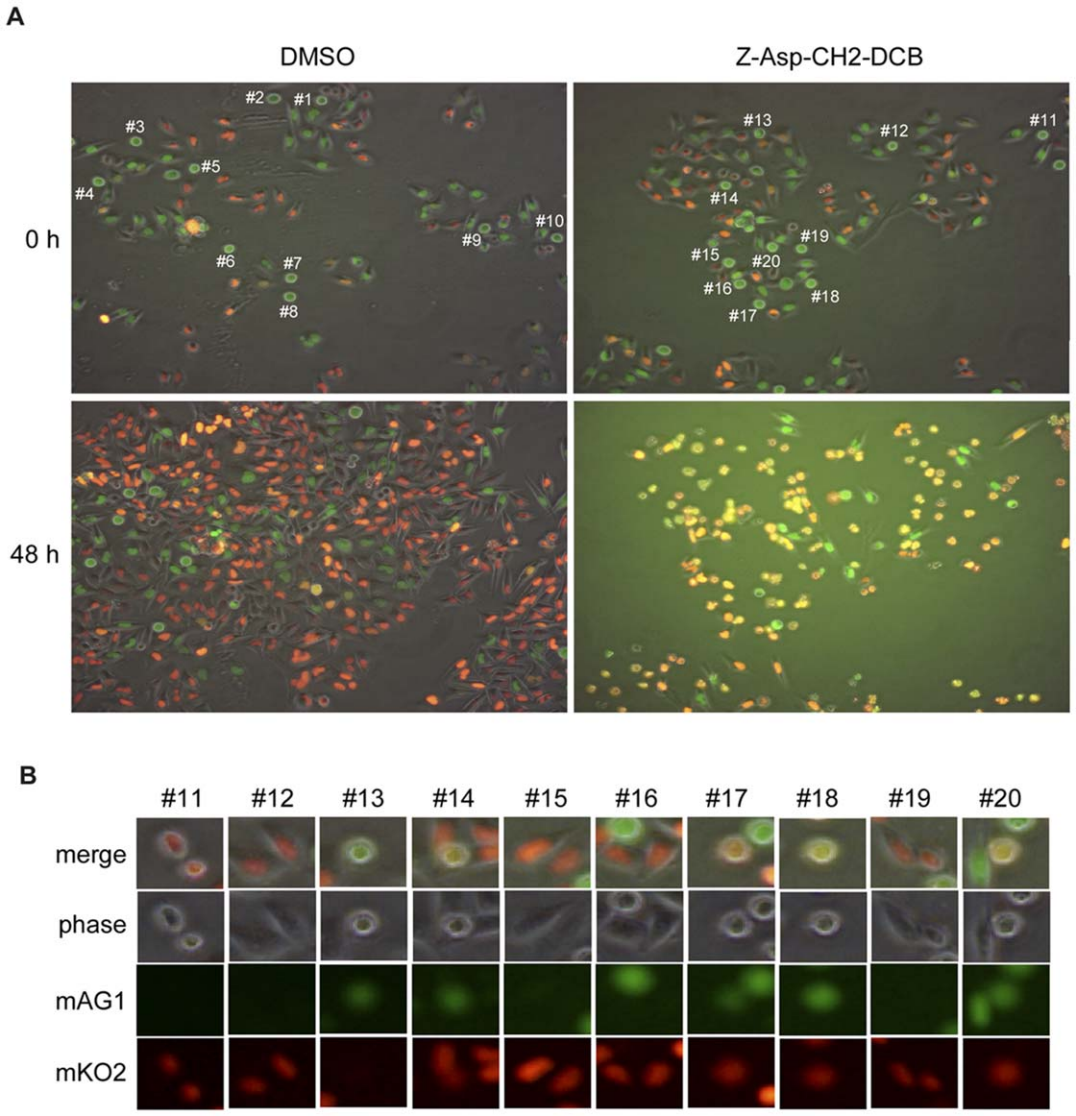
Peptide-based caspase inhibitor impairs the transition from M to G_1_ phases through inhibiting APC/C. **A**) HeLa.S-Fucci cells were cultured in the presence of 0.6% DMSO or 200 µM Z-Asp-CH_2_-DCB for 48 hours, and were observed with the time-lapse fluorescence microscope at 30-minute intervals (see Videos S6 and S7). The morphology of cells and the color of cell nuclei at 0 and 48 hours are represented. Ten mitotic cells with a round shape and a green color were randomly picked up from cells treated with DMSO (#1 to #10) and Z-Asp-CH_2_-DCB (#11 to #20). **B**) The merged images of the mitotic cells (#11 to #20 in **A**) at 5 hours after treatment with Z-Asp-CH_2_-DCB were shown by phase contrast, monomeric Azami-Green 1 (mAG1), and monomeric Kusabira-Orange 2 (mKO2) images, respectively.

To analyze the molecular basis for the effects of caspase inhibition during the cell cycle progression, HeLa.S-Fucci cells were used, which express monomeric Kusabira-Orange 2 (mKO2) and monomeric Azami-Green 1 (mAG1) fused to the ubiquitination domains of Cdt1 and geminin, respectively [31]. Since Cdt1 and geminin are the direct substrates of SCF^Skp2^ and APC/C^Cdh1^ complexes, respectively, the level of Cdt1 is highest at G_1_ phase whereas geminin is prominent during S, G_2_, and M phases [32]. Therefore, the cell nuclei of HeLa.S-Fucci cells during the cell cycle are labeled with orange of mKO2 fused to the ubiquitination domain of Cdt1 in G_1_ phase and green of mAG1 fused to the ubiquitination domain of geminin in S, G_2_, and M phases. We monitored the morphology of cells and the color of cell nuclei of HeLa.S-Fucci cells after the treatment with Z-Asp-CH_2_-DCB with the time-lapse fluorescence microscope for 48 hours (Figure 4A and Videos S6 and S7). Although the control cells treated with DMSO proliferated normally with oscillations in the fluorescence color changes (Figure 4A and Video S6), the number of caspase inhibitor-treated cells did not increase and a large fraction of cells showed the round shape with a yellow fluorescence (Figure 4A and Video S7). To further analyze the effects of the caspase inhibitor on the cell cycle progression at mitotic phase, ten mitotic cells which show a round shape with a green fluorescence were randomly picked up from the cells treated with DMSO (#1 to #10 in Figure 4A, upper left) and Z-Asp-CH_2_-DCB (#11 to #20 in Figure 4A, upper right), and the progression of mitotic phase of each cell was shown at intervals of 30 minutes for 5 hours (Figure S1). During the normal cell cycle progression from M phase to G_1_ phase, mKO2 fused to the ubiquitination domain of Cdt1 appeared after the disappearance of mAG1 fused to the ubiquitination domain of geminin and the execution of cytokinesis. Although 9 out of 10 cells treated with DMSO (#1–7, 9, and 10 in Figure S1) transited from M phase to G_1_ phase within 2.5 hours, a half of the cells treated with Z-Asp-CH_2_-DCB (#13, 14, 17, 18, and 20 in Figure S1) seemed to stay in M phase even at 5 hours later because these cells still showed a round shape. Surprisingly, 4 out of 5 cells (#14, 17, 18, and 20 in Figure S1) had a yellow fluorescence indicating the presence of both mKO2 and mAG1 fluorescent proteins in cells. To further confirm the coexistence of these fluorescent proteins in cells, the merged images of ten mitotic cells at 5 hours after the treatment with the caspase inhibitor (#11–20 in Figure S1) were shown with phase contrast and fluorescence of mAG1 and mKO2 (Figure 4B). The cells (#14, 17, 18, and 20) coexpressed both fluorescent proteins, suggesting that the failure of the cell cycle progression from M phase to G_1_ phase in the cells treated with the caspase inhibitor was caused by missing the activity of APC/C^Cdh1^ which is responsible for destruction of mAG1 fused to the ubiquitination domain of geminin at the transition from M phase to G_1_ phase.

**Figure 5:**
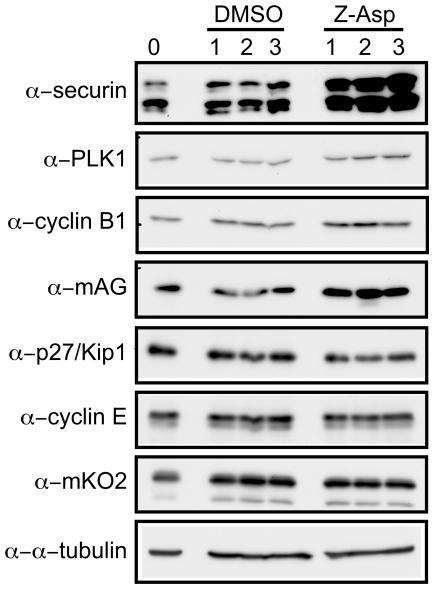
Substrates for APC/C are accumulated in caspase inhibitor-treated cells. HeLa.S-Fucci cells were cultured in the presence of 0.6% DMSO or 200 µM Z-Asp-CH_2_-DCB for the indicated times, and cells were harvested and lysates were subjected to immunoblot analysis with the antibodies as indicated.

If this is the case, target proteins for APC/C^Cdh1^ may be accumulated after treatment with caspase inhibitors. Therefore, we analyzed the protein levels of the substrates for APC/C and SCF complexes (Figure 5). The substrates for APC/C accumulated such as securin, PLK1, cyclin B1, and mAG1 fused to the ubiquitination domain of geminin, however, the protein levels of the substrates for SCF including p27, cyclin E, and mKO2 fused to the ubiquitination domain of Cdt1 did not change. These results suggest that the caspase inhibition attenuates the APC/C activities and leads the cells to arrest at mitotic phase.

## Discussion

We report here that the inhibition of caspase(s) resulted in the prevention of cell proliferation through cell cycle arrest at G_1_ and G_2_/M phases. The broad-spectrum caspase inhibitors, Z-Asp-CH_2_-DCB and Boc-Asp(Obzl)-CMK, were needed relatively at high concentrations for inhibition of cell proliferation, and thus there might be a possibility that the off-target effects of these compounds on, for example, other cysteine proteases are responsible for such inhibition [33], [34]. It could be concluded, however, that caspases such as caspase-3 and/or caspase-7 play a critical role in cell proliferation by the following reasons; (1) lower concentrations of the inhibitor specific to caspase-3 and -7 prevented cell proliferation (Figure 1B), (2) XIAP-BIR2, which is an inhibitory protein specific for caspase-3 and -7 through a mode of action different from that of the peptide-based inhibitors, prevented cell proliferation (Figure 2), (3) knockdown of caspase-7 using siRNAs and shRNA resulted in the inhibition of cell proliferation as well as cell cycle arrest at mitotic phase [22]. Although caspase-3 and -7 share a common substrate specificity and the available inhibitors unfortunately do not distinguish these two caspases, we speculate that the prevention of the cell cycle progression at mitotic phase by the caspase inhibitors is mainly due to the suppression of the caspase-7 activity because knockdown of caspase-3 using siRNA had no significant effect on cell proliferation [22].

Since caspase-3 and -7 are expressed as latent proenzymes, it is important to elucidate how these caspases, especially caspase-7, are activated during cell cycle. Caspase-8 and -9 are suggested to function as upstream enzymes to cleave and activate caspase-3 and -7 in apoptotic cells [1]–[3], and it is reasonable to ask whether the caspase activation machinery in apoptosis is also involved in the progression of cell cycle. Caspase-9, however, does not appear to function as an upstream caspase for caspase-3 and -7 during the cell cycle progression, because neither the peptide-based caspase-9 inhibitor nor XIAP-BIR3 prevented the cell proliferation (Figure 1B and Figure 2). Caspase-8 may contribute to the cell cycle regulation through activation of caspase-3 and/or caspase-7, since the peptide-based caspase-8 inhibitor prevented, even weakly, the cell proliferation (Figure 1B).

Caspase-3-/- mice are born in lower frequencies, and are smaller in size than their littermates [35], whereas caspase-7-/- mice are born without apparent abnormalities [36]. Double knockout mice lacking both caspase-3 and -7 die immediately after birth, although caspase-3/-7-deficient cells derived from the double knockout mice can be cultured and proliferate *in vitro* [36]. These results might suggest that caspase-3 and -7 are important for survival but not essential for the cell proliferation or cell cycle regulation. However, it has been reported that a deficiency in caspase-3 can induce activation of caspase-6 and caspase-7, and that in caspase-9 can enhance caspase-2 and caspase-6 in knockout mice [37]. In addition, the depletion of caspase-3 by siRNA induces a compensatory elevation in the caspase-7 level [38]. Therefore, the cells deficient in caspase-3 and -7 would not necessarily be expected to show an impairment of cell proliferation because of the compensatory functions of other caspases, that are not observed in the wild-type cells upon the treatment with the inhibitors of caspase-3 and -7.

Among the eight mammalian IAPs, XIAP is the only IAP that is a potent, direct inhibitor of the proteolytic activity of caspases and is able to inhibit caspase-3 and caspase-7 with nanomolar activity. Therefore, we tried to examine the effects of full length XIAP on cell proliferation. However, the cells overexpressing XIAP resulted in cell death during 24 hours' observation (data not shown). Since XIAP has multiple functions other than caspase inhibition, such as ubiquitin-E3 and NEDD8-E3 ligases [4], [5], [39]–[41], it may be essential for cell proliferation to maintain the protein level of XIAP in cells. We showed that the BIR2 domain of XIAP fused to the DsRed-monomer induced cell cycle arrest (Figure 2). Since this system is artificial and the endogenous XIAP protein level was not changed during cell cycle progression (data not shown), it is not clear whether XIAP itself contributes to the cell cycle progression through the inhibition of caspase activities. Although several lines evidences suggested that XIAP is involved in the cell cycle regulations at mitotic phase through the interaction with Chk1 and the degradation of survivin [42], [43], it is necessary to evaluate the possible contribution of XIAP to the cell cycle regulation at mitotic phase in relation to caspases.

Since activation of caspases by proteolytic cleavage is an irreversible reaction, there are several mechanisms for inhibiting spontaneous caspase activation to prevent apoptotic cell death during cell cycle progression. IAPs such as cIAP1, cIAP2, and XIAP function not only as caspase inhibitors but also as E3 ubiquitin ligases [4], [5], [39], [40]. Recently, it was reported that XIAP functions as a NEDD8-E3ligase for caspase-7 to prevent its proteolytic activity [41]. Therefore, IAPs may function as regulators for caspases during cell cycle progression by their activities as caspase inhibitors and/or E3 ligases for ubiquitin and NEDD8. Our data suggested that the transient activation of caspase-3 and/or caspase-7 around mitotic phase. This activation may be partially explained by the cell cycle-dependent relocalization of IAPs such as cIAP1 that localized in nuclei during interphase, late anaphase, and telophase while in the cytoplasm early in mitosis [44].

Cell cycle is controlled by the ubiquitin-mediated proteolysis, and the APC/C and SCF complexes function as E3 ligases that mark a variety of proteins with ubiquitin in a cell cycle-dependent manner [6]. The APC/C^Cdh1^ complex is active in the late M and G_1_ phases, and ubiquitinates various proteins such as Cdc20, Cdh1, Aurora A, Aurora B, PLK1, and geminin, leading to their proteolytic degradation [7], [8]. The results described in Figures 4, 5, and S1, in which the activities of not only APC/C^Cdh1^ but also APC/C^Cdc20^ were impaired, may indicate that a failure occurred in destruction of the cell cycle regulators in the caspase inhibitor-treated cells, that should disappear during these periods. Since these abnormalities were caused by the broad-spectrum caspase inhibitor, the subtype(s) of caspases contributing to the regulation of APC/C is unclear. Caspase-7 may be the most probable candidate, because shRNA specific for caspase-7 inhibited the progression from M to G_1_ phases [22]. The identification of the critical caspase-7 targets affecting the activities of APC/C and the development of caspase-7 specific inhibitors will provide clues to address these issues.

It was reported that caspases, including caspase-3, -7, -8, and -9, are constitutively activated in some human tumor cells [19]. Consistently, we observed a critical role of caspases for cell proliferation in HepG2, HeLa, and Jurkat cells in an apoptosis-independent manner. On the other hand, many caspase substrates that function in cell cycle checkpoints have been identified, and dysfunction of these proteins has been suggested to contribute to tumorigenesis [45]–[47]. For example, the Cdk inhibitors p21 and p27, cyclin E, and Rb regulate the transition of cell cycle from G_1_ to S phases, and Bub1, BubR1, Scc1/Rad 21, CENP-C, and INCENP are involved in the M phase progression [48]–[50]. Therefore, caspases activated in tumor cells may contribute to loss of cell cycle checkpoints and facilitate the rapid proliferation of these tumor cells. Since we observed that caspase inhibition induced cell cycle arrest at G_2_/M as well as G_1_ phases (Figure 3), caspase(s) may contribute to the cell cycle regulation during G_1_ phase in addition to M phase. It is thus interesting to evaluate a possible role of apoptosis-independent caspase activation and the following cleavage of their substrates during tumorigenesis.

## Materials and Methods

### Cell culture and transfection

HepG2 (a hepatocellular carcinoma line) and Jurkat (a leukemia T-cell line) cells were cultured in RPMI 1640 medium supplemented with 10% fetal bovine serum (FBS). HeLa (a cervical carcinoma line, clone D98AH2), HeLa-H2B-GFP [29] and HeLa.S-Fucci [31] cells were cultured in DMEM supplemented with 10% FBS. Transfection was performed using FuGENE 6 (Roche). According to the manufacturer's instructions, we optimized transfection conditions such as the ratio of the FuGENE 6 reagent to DNA and the incubation time in the serum-free medium. In our transfection conditions, we usually observed some apoptotic cells during the transfection process, which may be induced by some stresses such as the cytotoxicity of FuGENE 6 and the serum starvation. The culture medium contained the final concentration of DMSO at 0.6%, when cells were treated with the reagents as indicated in each experiments.

### Cell proliferation assay

Cell proliferation reagent WST-1 (Roche) was used to monitor cell viability according to the manufacturer's instructions. In brief, HepG2, HeLa, or Jurkat cells were plated in a 96-well plate at 4×10^3^, 2×10^3^, or 4×10^3^ cells, respectively, per well with 100 µl medium and incubated for 24 hours. After addition of caspase inhibitors, Z-Asp-CH_2_-DCB (Peptide Institute), Boc-Asp(Obzl)-CMK, and Ac-AAVALLPAVLLALLAP-DEVD-CHO, -LEHD-CHO, and -IETD-CHO (Calbiochem), cells were incubated for indicated days, followed by addition of WST-1 reagent to medium and further incubation for 2 hours. The cleavage of the tetrazolium salt WST-1 by mitochondrial dehydrogenases was determined by measuring the absorbance at 450 nm with a reference wavelength of 690 nm.

### Plasmid construction

The XIAP cDNA fragment was amplified from a human thymus cDNA library with the primers, 5′-GCCTCGAGAGATGACTTTTAACAGTTTT-3′ and 5′-GCGAATTCGATTAAGACATAAAAATTTT-3′, and cloned into the *Xho*I-*Eco*RI site of pBluescript SK(-) (Stratagene). The DNA sequence of the isolated XIAP was confirmed by the dideoxy chain-termination method. The fragments of XIAP-BIR2 with the linker region (amino acids 124 to 237) and XIAP-BIR3 (amino acids 252 to 350) were amplified with the primers, 5′-GCCTCGAGGCAGAGATCATTTTGCC-3′ and 5′-GCGAATTCTTAAATATTAAGATTCCG-3′, and 5′-GCCTCGAGCAAATTCAACAAATCTT-3′ and 5′-GCGAATTCTTACTCAAGTGAATGAGT-3′, respectively. The PCR method employing mutagenic oligonucleotide primers was used to generate D148A/E219R/H223V mutations in BIR2 and E314S mutation in BIR3. The resulting fragments containing the wild type (Wt) and mutated XIAP-BIR2 with the linker region and XIAP-BIR3 were sequenced and cloned into the *Xho*I-*Eco*RI site of pDsRed-Monomer-C1 (BD Biosciences) to generate pDsRed-XIAP-BIR2-Wt, pDsRed-XIAP-BIR2-3M containing D148A/E219R/H223V mutations, pDsRed-XIAP-BIR3-Wt, and pDsRed-XIAP-BIR3-1M containing E314S mutation, respectively.

### Immunoblot analysis

Cells were lysed in lysis buffer [20 mM Tris-HCl (pH 7.5), 150 mM NaCl, 1% Nonidet P-40, 50 µg/ml phenylmethanesulfonyl fluoride, 5 mM EDTA]. Protein samples were separated by SDS-polyacrylamide gel electrophoresis and blotted onto Immobilon polyvinylidene difluoride membrane (Millipore). Each protein was detected using primary antibodies as indicated, horseradish peroxidase-conjugated secondary antibodies, and ECL-plus detection reagent (GE Healthcare). Anti-DsRed polyclonal antibody (8376-1) and anti-p27/Kip1 monoclonal antibody (554069) were obtained from BD Biosciences; anti-Securin/PDS-1 monoclonal antibody (K0090-3), anti-Cyclin B1 monoclonal antibody (K0128-3), anti-monomeric Kusabira-Orange 2 polyclonal antibody (PM051), and anti-monomeric Azami-Green 1 polyclonal antibody (PM052) were from Medical & Biological Laboratories; anti-PLK1 rabbit monoclonal antibody (#4513) was from Cell Signaling; anti-cyclin E monoclonal antibody (sc-247) was from Santa Cruz Biotechnology; anti-α-tubulin monoclonal antibody (T6074) was from Sigma.

### Time-lapse fluorescence microscopy

For time-lapse fluorescence microscopy, HeLa-H2B-GFP and HeLa.S-Fucci cells were plated on a 35-mm glass bottom dish. The medium was replaced with MEM supplemented with 10% FBS without phenol red, and dishes were placed in a humidified chamber at 37°C that was mounted on a fluorescence microscope (model BZ-8000; Keyence) with a constant supply of mixed air containing 5% CO_2_. Cells were observed and image data were obtained automatically [22].

### Flow cytometry

HeLa cells (1×10^6^ cells) were incubated with 0.5 ml of propidium iodide/RNase buffer (BD Bioscience) after fixation with 70% ethanol for 1 hour at -20°C, and DNA content was measured using a FACSCaliber (Becton-Dickinson). 2×10^4^ events were analyzed for each sample, and data were plotted using Modifit software, and cell cycle profiles were determined by CELLQuest (Becton-Dickinson).

## Supporting Information

Figure S1 **Progression of mitotic phase of HeLa.S-Fucci cells treated without or with peptide-based caspase inhibitor.** HeLa.S-Fucci cells were cultured in the presence of 0.6% DMSO or 200 µM Z-Asp-CH_2_-DCB, and ten mitotic cells with a round shape and a green color were randomly picked up from cells treated with DMSO (#1 to #10) or Z-Asp-CH_2_-DCB cells (#11 to #20) as described in Figure 4A. The progression of the mitotic phase of each cell was observed with the time-lapse fluorescence microscope at 30-minute intervals for 5 hours.
(TIF)

Video S1 **Time-lapse observation of the effects of the expression of DsRed-monomer.** This video shows a typical example of the effects of the expression of DsRed-monomer (red) in HeLa-H2B-GFP cells in which chromatin is labeled with GFP (green). Image data were obtained automatically every 15 minutes for a period of 24 hours. The effects of DsRed-monomer expression were summarized in Figure 2C (Quick Time; 0.65 MB).
(MOV)

Video S2 **Time-lapse observation of the effects of the expression of DsRed-XIAP-BIR2-Wt.** This video shows a typical example of the effects of the expression of DsRed-XIAP-BIR2-Wt (red) in HeLa-H2B-GFP cells in which chromatin is labeled with GFP (green). Image data were obtained automatically every 15 minutes for a period of 24 hours. The effects of DsRed-XIAP-BIR2-Wt expression were summarized in Figure 2C (Quick Time; 0.86 MB).
(MOV)

Video S3 **Time-lapse observation of the effects of the expression of DsRed-XIAP-BIR2-3M.** This video shows a typical example of the effects of the expression of DsRed-XIAP-BIR2-3M (red) in HeLa-H2B-GFP cells in which chromatin is labeled with GFP (green). Image data were obtained automatically every 15 minutes for a period of 24 hours. The effects of DsRed-XIAP-BIR2-3M expression were summarized in Figure 2C (Quick Time; 0.88 MB).
(MOV)

Video S4 **Time-lapse observation of the effects of the expression of DsRed-XIAP-BIR3-Wt.** This video shows a typical example of the effects of the expression of DsRed-XIAP-BIR3-Wt (red) in HeLa-H2B-GFP cells in which chromatin is labeled with GFP (green). Image data were obtained automatically every 15 minutes for a period of 24 hours. The effects of DsRed-XIAP-BIR3-Wt expression were summarized in Figure 2C (Quick Time; 0.96 MB).
(MOV)

Video S5 **Time-lapse observation of the effects of the expression of DsRed-XIAP-BIR3-1M.** This video shows a typical example of the effects of the expression of DsRed-XIAP-BIR3-1M (red) in HeLa-H2B-GFP cells in which chromatin is labeled with GFP (green). Image data were obtained automatically every 15 minutes for a period of 24 hours. The effects of DsRed-XIAP-BIR3-1M expression were summarized in Figure 2C. (Quick Time; 0.94 MB)
(MOV)

Video S6 **Time-lapse observation of the effects of 0.6% DMSO in cell culture.** This video shows the culture of HeLa.S-Fucci cells in the presence of 0.6% DMSO. Image data were obtained automatically every 30 minutes for a period of 48 hours. The time course of the video indicates that 0.6% of DMSO did not have a significant effect on cell proliferation of HeLa.S-Fucci cells as described in Figures 4 and S1. (Quick Time; 1.9 MB)
(MOV)

Video S7 **Time-lapse observation of the effects of the caspase inhibitor in cell culture.** This video shows the effects of 200 µM Z-Asp-CH_2_-DCB on the morphology of cells and color of cell nuclei (green and orange) of HeLa.S-Fucci cells. Image data were obtained automatically every 30 minutes for a period of 48 hours. The effects of the caspase inhibitor were described in Figures 4 and S1. (Quick Time; 1.3 MB)
(MOV)
