# Supplementary figures and images for: Contribution of Caspase(s) to the Cell Cycle Regulation at Mitotic Phase
Source: PLoS One. 2011 Mar 30;6(3):e18449. doi: 10.1371/journal.pone.0018449 (PMC3068168; doi:10.1371/journal.pone.0018449)

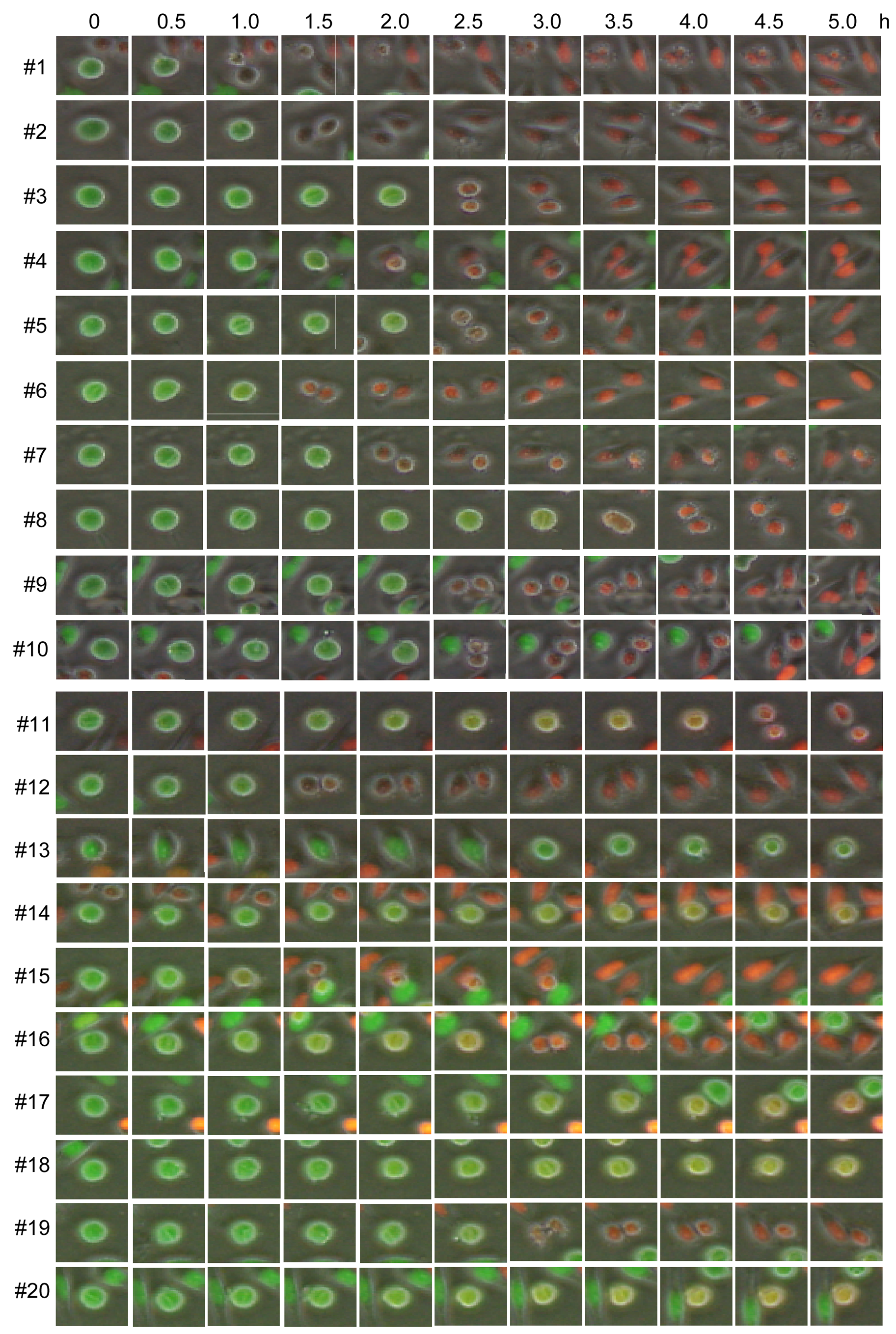

Supplement: Figure S1 — Progression of mitotic phase of HeLa.S-Fucci cells treated without or with peptide-based caspase inhibitor. HeLa.S-Fucci cells were cultured in the presence of 0.6% DMSO or 200 µM Z-Asp-CH2-DCB, and ten mitotic cells with a round shape and a green color were randomly picked up from cells treated with DMSO (#1 to #10) or Z-Asp-CH2-DCB cells (#11 to #20) as described in Figure 4A. The progression of the mitotic phase of each cell was observed with the time-lapse fluorescence microscope at 30-minute intervals for 5 hours. (TIF) [file pone.0018449.s001.tif]
